# Role of Cathepsins in *Mycobacterium tuberculosis* Survival in Human Macrophages

**DOI:** 10.1038/srep32247

**Published:** 2016-08-30

**Authors:** David Pires, Joana Marques, João Palma Pombo, Nuno Carmo, Paulo Bettencourt, Olivier Neyrolles, Geanncarlo Lugo-Villarino, Elsa Anes

**Affiliations:** 1 Research Institute for Medicines, iMed-ULisboa, Faculty of Pharmacy, Universidade de Lisboa, Portugal; 2 Instituto de Medicina Molecular, Faculdade de Medicina da Universidade de Lisboa, Portugal; 3 Centre National de la Recherche Scientifique, Institut de Pharmacologie et de Biologie Structurale, Toulouse, France; 4 Institut de Pharmacologie et de Biologie Structurale, Université de Toulouse, Université Paul Sabatier, Toulouse, France

## Abstract

Cathepsins are proteolytic enzymes that function in the endocytic pathway, especially in lysosomes, where they contribute directly to pathogen killing or indirectly, by their involvement in the antigen presentation pathways. *Mycobacterium tuberculosis* (MTB) is a facultative intracellular pathogen that survives inside the macrophage phagosomes by inhibiting their maturation to phagolysosomes and thus avoiding a low pH and protease-rich environment. We previously showed that mycobacterial inhibition of the proinflammatory transcription factor NF-κB results in impaired delivery of lysosomal enzymes to phagosomes and reduced pathogen killing. Here, we elucidate how MTB also controls cathepsins and their inhibitors, cystatins, at the level of gene expression and proteolytic activity. MTB induced a general down-regulation of cathepsin expression in infected cells, and inhibited IFNγ-mediated increase of cathepsin mRNA. We further show that a decrease in cathepsins B, S and L favours bacterial survival within human primary macrophages. A siRNA knockdown screen of a large set of cathepsins revealed that almost half of these enzymes have a role in pathogen killing, while only cathepsin F coincided with MTB resilience. Overall, we show that cathepsins are important for the control of MTB infection, and as a response, it manipulates their expression and activity to favour its intracellular survival.

Tuberculosis (TB) remains a worldwide health problem with 8 million new cases diagnosed and more than 1 million deaths per year, as reported by the World Health Organization^1^. The emergence of multi- (MDR) or extensively drug-resistant (XDR) strains of *M. tuberculosis* (MTB), the etiologic agent of TB, has brought renewed attention to the dangers of TB spread with the reports estimating 450,000 new cases of MDR-TB annually^1^. New strategies that, synergistically fight the disease through both antibiotic treatment and by enhancing the natural ability of the immune system to tackle the pathogen, might facilitate improved MTB clearance and thereby reduce the probability of generating resistant strains.

One of the first encounters of the immune system with the pathogen begins in the lungs where macrophages internalize the bacteria^2^. These cells are usually able to destroy bacteria upon phagocytosis and exposure to oxidative stress at an early stage, and subsequent acidification of the bacteria-containing phagosome upon fusion with late endosomes and lysosomes; there, the bacteria still encounter a toxic environment characterized mainly by the activity of proteolytic and lipolytic enzymes^3^. These events will lead to pathogen destruction and processing of its antigens to be presented to lymphocytes through the class II antigen presentation machinery. Pathogenic mycobacteria, however, impair this process by blocking phagosome maturation and consequent fusion with late endosomes and lysosomes, avoiding contact with their degradative enzymes^4^,^5^,^6^,^7^. Despite this capacity, there is evidence that a fraction of phagosomes still become fully mature to process and present mycobacteria antigens to lymphocytes^8^,^9^. In addition, the arrest of phagosome maturation by MTB may be overcome by macrophage activation through exposure to pro-inflammatory cytokines^10^,^11^,^12^ and signalling lipids^13^,^14^,^15^,or through activation of other cellular processes such as autophagy and apoptosis^10^,^16^,^17^,^18^, leading to the digestion of the pathogen. Collectively, this infers an important role for lysosomal effectors.

Cathepsins (Cts) are the most investigated class of proteases^19^. They operate in several cell functions such as protein processing, pathogen killing, antigen presentation, apoptosis and tissue remodelling^20^. Some cathepsins, such as cathepsins B, D, G and S are already known to interact and contribute to killing invading microorganisms^21^,^22^,^23^,^24^,^25^. There is also evidence for the role of cathepsins S, F, L and V in antigen presentation by processing the invariant chain linked to class II HLA molecules in several types of cells^26^,^27^,^28^,^29^,^30^. The type of cell infected and its stimulation also results in different kinetics of cathepsin activity. Resting macrophages (hereafter referred to as M0), for example, are thought to be prone to initiate a strong proteolytic activity upon microbial invasion, leading to the destruction of protein antigens. By contrast, interferonγ (IFNγ)- activated macrophages (hereafter referred to as M1) rely on control of acidification and on cystatin (natural inhibitors of cathepsins) expression to reduce this proteolytic response in order to preserve epitopes, and thus elicit more efficient lymphocyte priming^3^,^31^,^32^,^33^,^34^.

We previously showed that a direct consequence of mycobacteria inhibition of nuclear factor- κB (NF-κB) activity is the impairment of the delivery of lysosomal enzymes to phagosomes, which results in reduced pathogen killing^14^. It can therefore be argued that one of the best-characterized survival mechanisms of MTB is the avoidance of contact with active cathepsins by inhibition of phagosome maturation. The other mechanism of preventing direct digestion by cathepsins is when bacilli escape from the phagosome into the cytosol, leading to inflammasome activation and subsequent cathepsin B-dependent pyroptosis or pyronecrosis^35^,^36^,^37^. These evasion mechanisms allow for replication and/or spread of the bacilli to neighbour cells. Yet, it is not clear if MTB controls cathepsin activity or whether it avoids contact with the compartment(s) where cathepsins become active.

In this study we screened for how cathepsins and their inhibitors cystatins are regulated at the level of gene expression in primary human macrophages during infection with MTB, as well as with *M. smegmatis*. The latter is a non-pathogenic mycobacterium that is readily killed in macrophages and that shares a significant number of orthologous genes with MTB^38^, making it suitable control for the comparison and identification of molecules involved in the pathogenesis of TB^39^. Our results show that MTB induces a general down-regulatory profile of cathepsin expression within macrophages. This was associated with a concomitant decrease in cathepsin protein levels and enzymatic activity, favouring an increased intracellular survival of the pathogen.

## Results

### Cathepsin and cystatin expression is modulated by IFNγ stimulation

In order to determine the role of cathepsins and their natural inhibitors during mycobacterial infection, we started by performing qRT-PCR transcriptomic analysis of cathepsins and cystatins expressed in macrophages at early stages of infection. We hypothesized that if the infection translates into altered cathepsin expression, those alterations would be measurable as early as 24 h post-infection since otherwise their impact during infection would be less significant. In fact, in another gene expression screen, Tailleux and colleagues^40^ already pointed that, while overall gene expression levels change throughout the course of infection, the majority of host genes showed regulation induced by MTB in the first 24 h and remain unchanged thereafter. Furthermore, at an early time point such as 24 h, the extent of MTB-induced cell death is minimal (Fig. S1), and thus eases the interpretation of the data.

**Figure 1:**
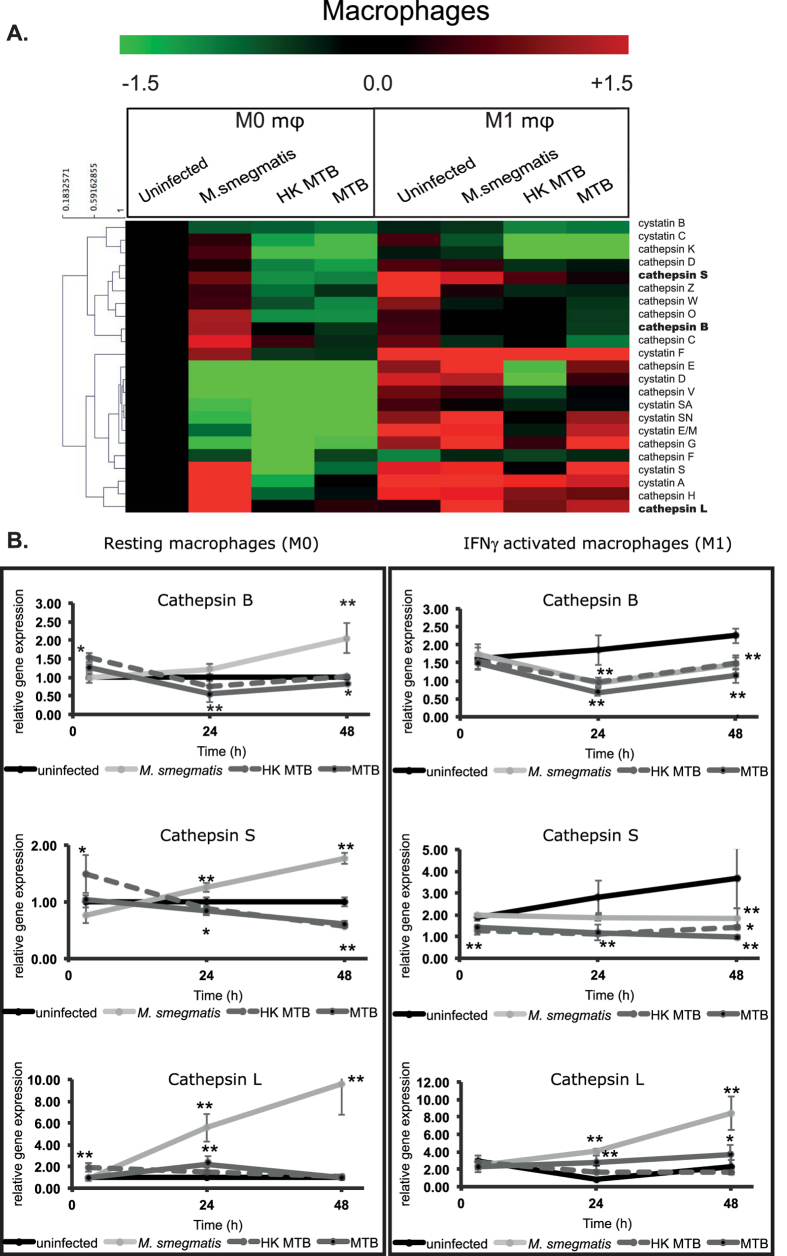
Gene expression of cathepsins and cystatins during infection of macrophages by MTB, HK MTB and *M. smegmatis*. (**A**) Heatmap of qRT-PCR quantification of mRNA obtained from macrophages after 24 h of infection. Values are depicted as log_2_ gene expression relative to uninfected macrophages. (**B**) Gene expression of cathepsin B, S and L in M0 or M1 macrophages infected with MTB, HK MTB or *M. smegmatis* along 48 h. Values are depicted relative to uninfected control (*p < 0.05 and **p < 0.01 relative to uninfected control; n = 5).

In our analysis, we focused on macrophages infected with MTB and compared them to non-infected cells or cells with phagocytosed heat-killed MTB (HK MTB), and to macrophages infected with the non-pathogenic *M. smegmatis*. For the host cells, we used M0 and M1 macrophages to mimic the different states of macrophage activation that MTB encounters, including those bystander macrophages that become exposed to the effects of pro-inflammatory cytokines. As shown in Fig. 1A, we observed that stimulation with IFNγ led to an up-regulation of the majority of screened cathepsins and cystatins, indicating a clearly distinguishable cathepsin profile in M1 macrophages relatively to M0 ones. The increase on gene expression observed for the majority of cystatins, suggests that although there is an increased cathepsin gene expression, a concomitant increment of their inhibitors should impair their activity in M1 macrophages. The exceptions to this global up-regulation were cathepsins F, L, K and cystatin B; cathepsin F was the strongest observation of down-regulation after IFNγ stimulation.

### *M. tuberculosis* infection induces the down-regulation of the majority of cathepsins and cystatins

Next, we compared macrophages infected with live MTB either to internalized heat-killed MTB or those infected with *M. smegmatis* in order to highlight the different regulatory phenotypes that they induce and potentially correlate them with MTB pathogenicity, or simply to discard them as general phagocytosis phenomena.

As shown in Fig. 1A the infection of M0 macrophages with MTB or *M. smegmatis* resulted in different profiles of cathepsin expression, specific for each species. MTB infection of macrophages results in the most prominent regulation, leading to a down-regulation of the majority of analysed cathepsins and cystatins, with the exception of cathepsins H, L and cystatin A, when compared to uninfected cells. HK MTB also induced a very similar down-regulation, while *M. smegmatis* infection resulted in the up-regulation of the majority of cathepsins (Fig. 1A). By contrast, the expression of the majority of cystatins was decreased in all conditions as compared to uninfected cells (Fig. 1A). In the case of M1 macrophages, although IFNγ treatment increased the expression of most cathepsins in uninfected macrophages, we observed a global down-regulatory expression profile for these enzymes in these cells when infected with MTB (Fig. 1A). When comparing uninfected and MTB-infected M1 macrophages, together with cystatin C, cathepsins B, C, F, K, L, S, W and Z were the most differentially expressed. A comparison of M1 macrophages challenged *with M. smegmatis* and those infected with MTB revealed that, while the expression of most cathepsins were down-regulated in the TB context, cathepsin E, F, G and V remained unaltered (Fig. 1A). Moreover, the expression levels of cathepsins B, C, H, O, S and L were the most differentially regulated between the two mycobacteria; in the case of cystatins, the most distinguishable were cystatins A, F and S. Interestingly, cathepsin L showed the opposite effect, being up-regulated upon infection with MTB, similarly to that observed in M0 macrophages (Fig. 1A).

Cathepsins B, S and L are some of the most highly expressed cathepsins in the lysosomes of antigen-presenting cells, and are described as participating in diverse cell functions, such as antigen processing, TLR signalling and cytokine production^20^. These features should render them as obvious candidates to participate in MTB killing and antigen presentation. Our initial screen revealed that these three cathepsins were down-regulated in M0 macrophages (more hydrolytic) during MTB infection, relatively to *M. smegmatis* infection. For these reasons, we next analysed the mRNA expression of these cathepsins at 3, 24 and 48 h post infection (Fig. 1B). In M0 macrophages (left panel), an increase of cathepsin B, S and L mRNA was observed during infection with *M. smegmatis* in contrast to cells challenged with HK MTB or MTB. In addition, while cathepsin B and S gene regulation has an opposite profile in MTB- and *M. smegmatis*-infected cells, the gene expression of cathepsin L was shown to be up-regulated in both conditions, albeit significantly higher in *M. smegmatis* infected cells (*P* < 0.001). In M1 macrophages, IFNγ had a distinct impact on the kinetics of cathepsin expression (Fig. 1B right panel). For cathepsins B and S, IFNγ induced an increase in expression at all time points relatively to M0 macrophages. During challenge with *M. smegmatis* or with MTB (or HK MTB), cathepsins B and S were down-regulated relatively to unchallenged M1 macrophages, and cathepsin L was up-regulated for all bacterial challenges compared to unchallenged M1 macrophages. For the latter cathepsin, its expression did not respond to IFNγ stimulus in uninfected macrophages.

All together, these results suggest that MTB induces a global down-regulation of cathepsin expression that may result to its advantage in human macrophages, a capacity that is lacking in the non-pathogenic *M.smegmatis*.

### *M. tuberculosis* infection reduces cathepsins B and S, but not L, protein levels and enzymatic activity

**Figure 2:**
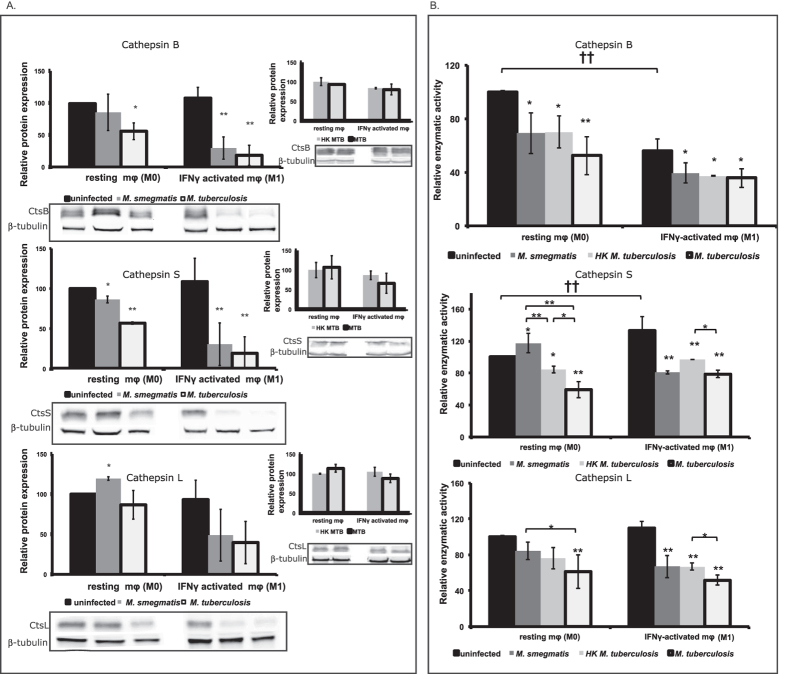
Protein expression and enzymatic activity of cathepsin B, S and L in M0 or M1 macrophages infected with MTB, HK MTB or *M. smegmatis* for 24 h. (**A**) Protein expression: Bar plots represent protein expression relative to uninfected control and were obtained by densitometry analysis of the western blots relative to the β-tubulin control. (**B**) Cathepsin activity was measured with a fluorimetric substrate that is activated by cleavage of a cathepsin-specific sequence. Values are depicted relative to uninfected control, and the statistical comparisons were performed within M0 or M1 groups relative to their respective uninfected control (*p < 0.05 and **p < 0.01; n = 4) or between groups (^†^p < 0.05 and ^††^p < 0.01; n = 4) unless otherwise indicated in the plot.

Given the important roles of cathepsins B, S and L, in antigen-presenting cells^20^, we next decided to deepen our analysis of these three enzymes. In order to understand their relevance in infection, we addressed their expression at the level of protein quantity and enzymatic activity. Our results show that in human macrophages, the protein expression for cathepsins B and S was lowered during infection with MTB, regardless of the activation state of the host cells (Fig. 2A). Moreover, the protein expression for both enzymes was significantly lowered during *M. smegmatis* and MTB infection of M1 macrophages (*P* < 0.01 and *P* = 0.026, respectively). For cathepsin L, no significant changes in protein levels were detected after infection with the two species of mycobacteria in M1 macrophages, but there was a slight increase in M0 macrophages after *M. smegmatis* infection (Fig. 2A bottom panel). When comparing MTB with HK MTB no differences in protein quantity were detected by Western-blot for the three cathepsins (Fig. 2A right panels).

Concerning cathepsin activity in human macrophages, there was a general reduction observed for the three cathepsins after infection with MTB (Fig. 2B). For cathepsin B, we noticed that its basal activity in M0 macrophages was a higher compared to M1 macrophages, and it was reduced similarly in these cells after challenged with both mycobacteria species (Fig. 2B top panel). For cathepsin S, while its basal activity was higher in M1 compared to M0 macrophages, it became differently regulated depending on the mycobacterial challenge (Fig. 2B, middle panel). Indeed, MTB infection significantly reduced cathepsin S activity in both type of macrophages. Yet, infection with *M. smegmatis* led to a significant increase of cathepsin S activity in M0 macrophages, but towards a decrease in M1 macrophages. For cathepsin L, there was no difference in the activity level in both type of macrophages, and only infection with live MTB led towards a significant activity decrease in these cells (Fig. 2B, bottom panel).

Collectively, these results not only confirm our observations at the mRNA level, but also evidence that MTB controls both the protein expression and activity level of cathepsins, in particular that of B, S and L.

### Pharmacological- or cystatin C-based inhibition of cathepsin activity increases MTB survival in macrophages

**Figure 3:**
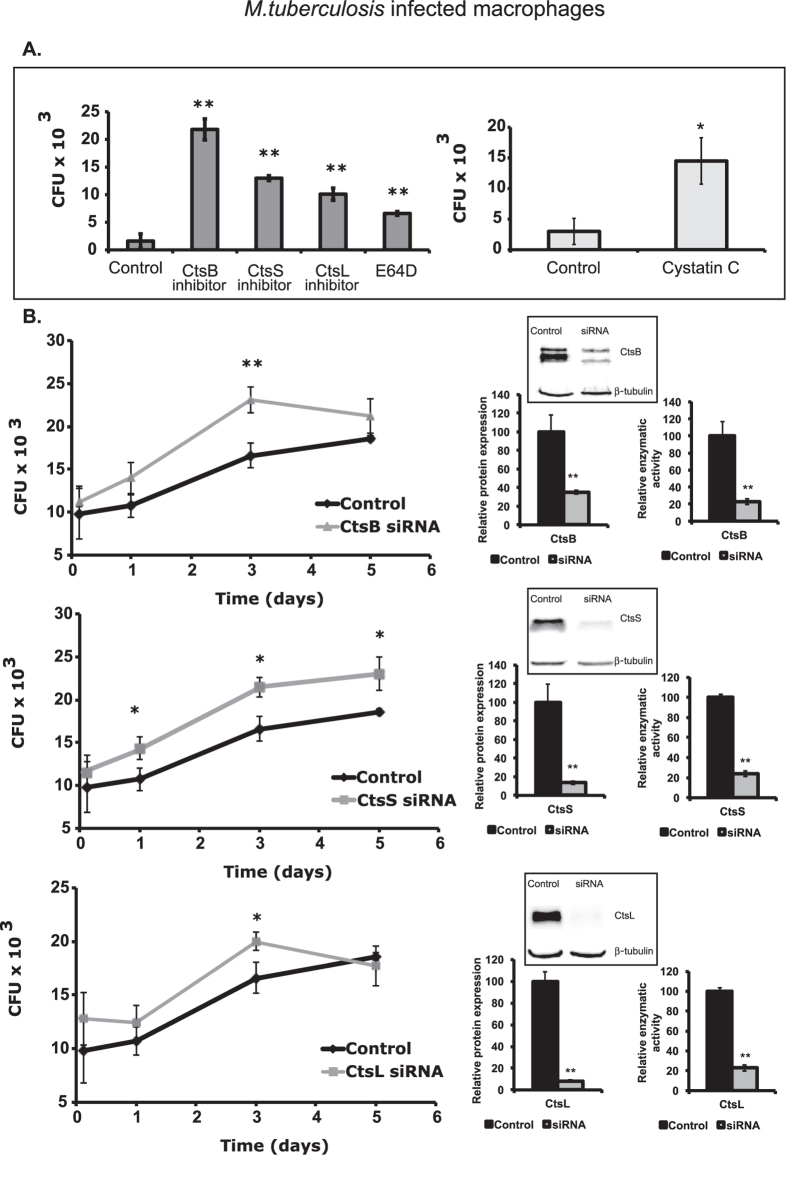
Effect of cathepsin B, S and L inhibition on MTB survival. (**A**) Cathepsin activity was inhibited using 20 μM of specific inhibitors or cystatin C 24 h prior to infection or 10 μM of the general cysteine cathepsin inhibitor E-64d after infection in M1 macrophages. Values are depicted relative to untreated control (*p < 0.5; **p < 0.01 relative to untreated control). (**B**) Effect of cathepsin B, S and L silencing on MTB survival in resting (M0) macrophages. Each cathepsin was silenced by siRNA 3 days prior to infection in order to achieve maximum protein silencing and reduction of enzymatic activity (on the right). Controls were transfected with a scramble siRNA (*p < 0.05; **p < 0.01 relative to control; n = 3).

Our results thus far led us to infer that MTB regulation of cathepsin expression and activity may be advantageous for its survival in human macrophages. To test this, our first approach was to use available chemical inhibitors to inactivate CtsB, CtsS and CtsL, and a general inhibitor of cysteine cathepsins E-64d^41^, and then measure the consequences on intracellular survival in macrophages. The intracellular bacteria load was similar after 3 h of bacteria internalization in all conditions tested (data not shown), indicated that treatment with the inhibitor did not affect the binding and uptake of MTB by macrophages (Fig. 3A). After 24 h post-infection, our results revealed that treatment with chemical inhibitors of cathepsins resulted in a significant increase in MTB intracellular survival (*P* < 0.001) in M1 macrophages, with a 10-fold increased survival when using CtsB inhibitors and approximately 5-fold increased survival when using CtsS and CtsL inhibitors (Fig. 3A, left). By contrast, the inhibitors failed to produce any significant results in M0 macrophages (data not shown). For this reason, we decided to treat these cells with cystatin C, the endogenous inhibitor of CtsB, CtsS and CtsL^42^. Our results showed that treatment with cystatin C led to a significant (*P* = 0.01) 5-fold increase of MTB survival after 24 h of infection in M0 macrophages (Fig. 3A right).

### siRNA-mediated gene silencing for cathepsins B, S and L results in increased survival of MTB in human primary macrophages

Our results with chemical inhibitors and cystatin C revealed the relevance of cathepsin activity for MTB killing. However, since we were limited to a 24 h analysis due to the cytotoxic effects of these inhibitors, we decided to employ a more selective inhibition of cathepsin activity by targeting their gene expression by siRNA as a second approach. We were able to achieve approximately 80% reduction in protein expression and cathepsin activity for cathepsins B, S or L using this technique in macrophages, which enabled us to analyse the full kinetic of MTB survival throughout 5 days of infection (Fig. 3B, right panels). Our results revealed that all three cathepsins are important for MTB control (Fig. 3B). While the most significant results occurred at day 3 post-infection for the inactivation of the three cathepsins, we observed a significant increase in MTB survival (*P* < 0.05) throughout the entire infection (Fig. 3B middle panel) in macrophages deficient for cathepsin S. Moreover, we performed flow cytometry analysis to determine if the differences observed were due to an increase of the internalization of bacteria and not due to increased survival. The results reveal similar bacterial load within macrophages after the 3 h of internalization (Fig. S2).

### Medium-throughput analysis of the effects of all cathepsins on MTB intracellular survival within human macrophages

**Figure 4:**
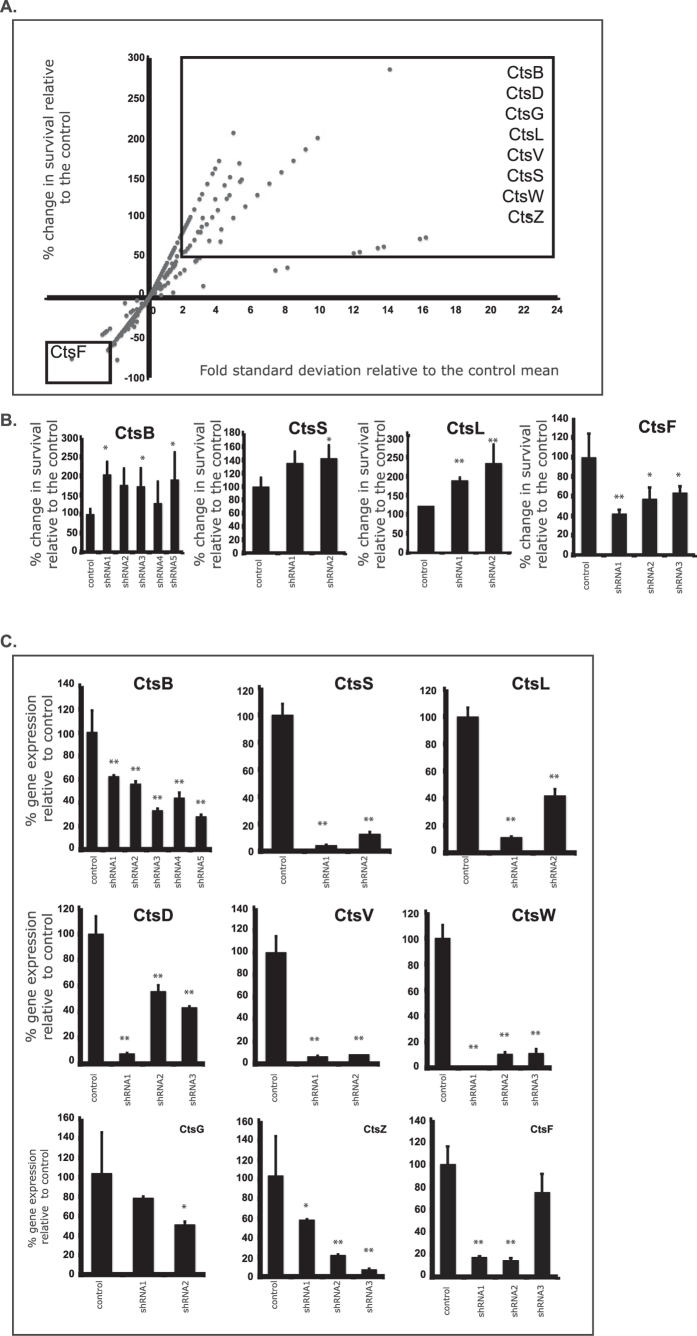
Medium-throughput analysis of the effects of cathepsins and cystatins on MTB intracellular survival within human macrophages. (**A**) Distribution of individual shRNA scores obtained from two independent screens. shRNAs were selected as “hits” (boxed dots) when there was an increase or decrease of MTB survival of >50%, and also of >2 standard deviations from the mean of the controls. (**B**) Quantification of the percentage of MTB survival within macrophages knocked down for cathepsins B, S, L and F. Only the shRNAs that produced stable puromycin-resistant cell lines are shown (*p < 0.05; **p < 0.01 relative to control; n = 5). (**C**) Quantification of cathepsin gene expression knock down in macrophages relative to control (*p < 0.05; **p < 0.01 relative to control; n = 4). Up to 5 shRNA lentivirus constructs were used per experiment.

The effects observed for the inhibition of cathepsin B, S and L on Mtb intracellular survival prompted us to extend our analysis to all cathepsins by using a siRNA methodology with a lentivirus library addressed to each individual cathepsin in THP1-derived macrophages. As shown in Fig. 4, of all cathepsins listed in Fig. 1A, the knock-down of Cts B, D, G, L, V, S, W and Z resulted in increased MTB survival within THP1 macrophages. Similarly to human primary macrophages, a decrease in Cts B, S and L resulted in increased pathogen survival in THP1 cells (Fig. 4B top panels). Surprisingly, the knock-down of CtsF revealed an opposite effect, leading to increased pathogen killing (Fig. 4B bottom panel). Control of the gene expression knock downs are depicted in Fig. 4C.

## Discussion

Lysosomal proteolysis is a crucial cell process for maintaining homeostasis. Proteolytic enzymes participate in the recycling of cell components, elimination of toxic molecules, processing of other host proteins and digestion of bacteria taken up by phagocytosis^19^. MTB is an intracellular parasite par excellence, and is able to persist by resisting within macrophages by avoiding the degradative mechanisms of the endocytic pathways. The state of macrophage activation during the initial steps of mycobacterium phagocytosis is of relevance for the type of immune response elicited. Resting macrophages (M0) are thought to be more prone to initiate a strong proteolytic activity upon microbial Phagocytosis, while IFNγ-activated macrophages (M1) rely on control of this proteolytic response in order to preserve epitopes for lymphocyte priming^3^,^31^,^32^,^33^,^34^. In this study, M1 macrophages showed a significant increase in the expression of several cathepsins, presumably related to their roles in antimicrobial killing and/or antigen presentation. The most notable exception was CtsF that was down-regulated upon macrophage activation. No changes on gene expression between M0 and M1 macrophages were observed for cathepsins L, K and O. Interestingly, Cts S and V, which were shown to be involved in antigen presentation^26^,^28^, were up-regulated upon IFNγ macrophage activation. Among those that were up-regulated, an increase in expression did not necessarily result in a higher proteolytic activity. Two major reasons can be attributed for a decrease of cathepsin hydrolytic activity in IFNγ-activated macrophages. The first is the result of pH alkalinisation due to the potent oxidative response of these cells^31^,^32^,^33^. CtsS is an exception, known to maintain its stability and activity even at neutral pH^43^. Beers and colleagues^44^ showed that IFNγ stimulation of macrophages results in increased processing of HLA class II-invariant chain complex. Our results are in agreement with this finding since we show that CtsS overall proteolytic activity increases by 40% in activated uninfected macrophages, thereby arguing that this cathepsin is a relevant contributor to proteolysis in a less acidic phagosome environment (Fig. 2B). The second reason is related to the exacerbated cystatin activity on M1 macrophages. Our results show that, with the exception of cystatin B, C and SA, all others protease inhibitors tested were up-regulated in IFNγ-activated macrophages. This surprising result again reinforces the idea that an increase in cathepsin expression may not be associated with an increased activity in activated macrophages.

The roles played by cathepsins in protein processing and digestion suggests that their activity might be important for MTB killing and for processing of its antigens. MTB improves its intracellular survival by inhibiting cellular processes that may lead to its destruction, such as phagosome maturation and fusion with lysosomes. Nevertheless, depending on the route of entry, recognition and external stimuli, a fraction of the MTB-phagosomes fully mature^8^,^45^,^46^, presumably leading to the recruitment and activation of lysosomal cathepsins. Moreover, some of these cathepsins are already active in early endosomes^47^, suggesting they may come in contact with MTB even when phagosome maturation is arrested.

A recent study demonstrated that MTB infection impairs the proteolytic activity of human macrophages^48^. The authors inferred this was probably due to a decreased acidification as a consequence of the induction of an M1 profile during infection and enhancement of the NADPH oxidase function^48^. We propose that one of the mechanisms that MTB uses to persist inside host cells is by avoiding the degradative action of lysosomal cathepsins through inhibition of their expression and/or impairment of their activity. Our results show that MTB infection of host macrophages results in a general down-regulation of cathepsin expression, when compared to the cathepsin expression profile elicited upon infection with the non-pathogenic *M. smegmatis*. The macrophage response to *M. smegmatis* included an overexpression of cathepsins B, C, K, L, O, S and H. CtsH has been detected in early endosomes and phagosomes of the mouse macrophage cell line J774^49^ and was found to become concentrated in late endosomes of mature DCs^50^, suggesting a role in antigen processing.

The major down-regulation of enzymes promoted by MTB, but not *M. smegmatis,* in macrophages included cathepsins B, D and S. CtsB and CtsD have been shown to regulate cell death in response to microbial infection by inducing pyroptosis in response to inflammasome activation (CtsB)^36^,^51^ or triggering apoptosis (CtsD)^22^. By inhibiting cell death during the first stages of infection, MTB is able to replicate in its intracellular niche, generating a higher burden of bacilli before erupting from a necrotic cell.

A strong decrease in cathepsins B, S and L activity was observed when M1 macrophages were infected either with MTB or *M. smegmatis*. This might be a general mechanism to reduce proteolysis in activated macrophages in order to preserve peptide antigens to be presented, such as the one reported by Savina and colleagues for dendritic cells trough NADH oxidase–mediated alkalinization of the phagosome^32^. However, Yates and colleagues^31^ described that, although this reduction in proteolysis might also be dependent on phagosomal alkalinisation, when activated macrophages were stimulated with LPS the phagosomes readily acidified. This indicates that another type of protease regulation. In fact, our results show that this regulation appears to occur at the protein level, since we observed a strong depletion of cathepsins B and S in MTB-infected IFNγ-stimulated macrophages. No significant protein reduction was detected for CtsL in our experiments. Yet, a reduction in cathepsin L protein levels was described by Nepal and colleagues^52^ in response to infection of IFNγ-stimulated mouse bone marrow macrophages with live or heat-killed MTB. Therefore, enhanced differences may be attributed to the type of host cells used. This is in line with the results by Podinovskaia *et al.*^48^ showing a decrease in hydrolytic activity during infection of human macrophages, but not in murine macrophages.

There are very few reports on the literature related to the involvement of cathepsins with mycobacteria survival. Cathepsin G, a serine protease was found to increase MTB survival in THP1 cells^24^. Another group reported that the programmed cell death induced by MTB was not dependent on caspase-1 or cathepsin B upon ESAT-6 expression^37^. Our group identified many lysosomal enzymes and membrane-trafficking regulators, including cathepsins that were regulated by NF-κB during infection. The direct consequence of NF-κB inhibition was the impaired delivery of lysosomal enzymes to mycobacterial phagosomes and reduced killing^14^.

Here, we show that, while *M. smegmatis* stimulates cathepsin expression, MTB down-regulates the expression of these enzymes. In addition, we found few differences in cathepsin regulation between infection with live or heat-killed MTB, suggesting the involvement of MTB components such as cell wall lipids or other effectors that could be released by either live or during heat inactivation of MTB to potentially modulate host cell functions and the surrounding tissue^53^,^54^. Although other bacterial effectors, such as those related to ESX secretory systems, are dependent on metabolic active bacteria^36^,^55^,^56^, there is evidence suggesting heat-killed MTB are able to prevent acidification and proteolytic activity within macrophage phagosomes^3^,^57^. Nevertheless, there were a few exceptions observed, for example, in M1 macrophages for which only live MTB induced an up-regulation of CtsG and cystatins S and E/M gene expression.

We wondered if this down-regulation would have a direct impact in the intracellular survival of MTB. We approached this problem by inhibiting cathepsin activity through a diverse set of techniques, using specific chemical inhibitors of CtsB, CtsS and CtsL, a general inhibitor of cysteine cathepsins E-64D, their natural inhibitor cystatin C, and also by silencing their gene expression with siRNA. All these methods converged upon the same conclusion; that is, inhibition of cathepsins B, S and L results in increased survival of MTB. It is still noteworthy that we could only obtain statistically significant results with the chemical inhibitors in activated macrophages. This might be due to the decrease in cathepsin activity we observed when infecting those cells. That effect, coupled with chemical inhibition would possibly produce a drastic ablation of cathepsin activity that could be more easily measured.

Interestingly, we found a down-regulation for cystatin C in M0 and M1 macrophages upon infection with both mycobacteria species (Fig. 1A). Since cystatin C is a natural inhibitor of cathepsins B, S and L, we expected a higher activity of these proteases. Yet, the major effect was observed for CtsB and S (Fig. 2B) but not for CtsL, which was the only one found to be up-regulated at the mRNA level (Fig. 1A). Nonetheless, inhibition with cystatin C resulted in a significant increase of MTB survival after 24 h of infection (Fig. 3A) in M1 macrophages. Moreover, individual siRNA silencing of Cts B, S and L also revealed an increase in MTB survival throughout 5 days of infection. Furthermore, the medium throughput assay that was performed to extend the silencing of a large set of cathepsins during MTB infection in THP1 cells confirmed the effect on MTB survival of Cts B, S and L, and provided new evidence for the role for cathepsins D, G, V, W and Z. Surprisingly, CtsF silencing induced more pathogen killing, a result we wish to further explore in the future. Altogether, our results consistently show that important role for cathepsins, and in particular for cathepsin B, S and L, for the intracellular control of MTB infection in human macrophages, and also the promising role of cystatins (natural inhibitors of cathepsins) during infection.

Establishment and progression of MTB remains somewhat of a mystery in humans. However, a deeper understanding of the early events in TB is essential to identifying new and effective strategies of preventing active state of this disease. Thus, we have elucidated some of the strategies that MTB uses to be able to survive and replicate inside the macrophage, as well as studied the conditions for which the host cell is able to control infection (as is the case of infection with *M. smegmatis*). We foresee the manipulation of Cts B, S and L activity as a viable strategy to help control the infection for the benefit of the patient. For this to happen, it is not enough to increase their expression and protein levels, as their activity is tightly controlled by cystatins, and for CtsB, by the acidification of the vacuole. An approach to address this issue is to target directly cystatin expression. Indeed, we observed an up-regulation of cystatins A, E/M, F, Cyst S and SN upon MTB infection of activated macrophages, suggesting the bacteria has already adapted this strategy in the arms race of host-microbe co-evolution in order to suppress cathepsin activity and enhance its survival fitness. Of particular interest, we predict that manipulation of CtsS, whose hydrolytic activity is less dependent on the pH, and CtsF, a cathepsin up-regulated during MTB infection and whose silencing leads to increased pathogen killing, will be an ideal starting point to test the true potential of cathepsin-based strategies to enhance the control of MTB infection.

In conclusion, cathepsins represent a very important part of the immune system and need to be properly kept under control to avoid pathological damage to cells or tissues. Due to their emerging role in activation of innate and adaptive immune responses, this study supports the notion that cathepsins and their inhibitors constitute attractive therapeutic targets. Along with other studies, we believe our findings contribute to basic but important knowledge for potential use in developing drugs that boost the innate immune system or targets bacterial virulence factors that interfere with cathepsin activity and are important for TB pathogenesis.

## Materials and Methods

### Cell lines and culture conditions

Human peripheral blood monocyte-derived macrophages (PBMCs) were obtained from buffy coats from healthy donors provided by the national blood institute (Instituto Português do Sangue, Lisbon, Portugal). Differentiation of the monocytes into macrophages proceeded as previously described^58^. When required, macrophages were stimulated with 100 IU/ml IFNγ, 18 h prior to infection. The human acute monocytotic leukemia cell line THP-1 (ATCC TIB202) was maintained as described previously^8^. Differentiation of THP-1 monocytes into macrophages was induced overnight with 20 nM phorbol 12-myristate 13-acetate (PMA). The strain *Mycobacterium smegmatis* mc2155, containing a p19 (long lived) EGFP plasmid, was kindly provided by Dr. Douglas Young (London School of Hygiene and Tropical Medicine, London, UK), and the green fluorescent protein (GFP)-expressing strain of *M. tuberculosis* (H37Rv-pEGFP) plasmid was a kind gift from G. R. Stewart (University of Surrey, United Kingdom). *M. smegmatis* was grown in medium containing Middlebrook’s 7H9 Medium (Difco), Nutrient broth (Difco) supplemented with 0.5% glucose and 0.05% Tween 80 at 37 °C on a shaker at 200 r.p.m^13^. MTB H37Rv was grown in Middlebrook’s 7H9 medium or 7H10 solid medium and supplemented with 10% OADC Enrichment (Difco)^13^.

### Macrophage Infection

Bacterial cultures on exponential grown phase were centrifuged, washed in phosphate-buffered saline (PBS). Bacteria were then re-suspended in the desired culture medium without antibiotics. In order to dismantle bacterial clumps, the bacterial suspension was passed through a 21 G needle followed by 5 min ultrasonic bath. Residual clumps were removed by 1 minute centrifugation at 500 × *g*. Single-cell suspension was verified by fluorescence microscopy. When required, bacteria were heat-killed by incubation at 80 °C for 20 min prior to phagocytosis. Bacteria viability was confirmed by platting HK treated bacteria in appropriate medium following incubation at 37 °C for three weeks. Macrophages were infected with an MOI of 1 for 3 h at 37 °C with 5% CO_2_. Following internalization, cells were washed three times with PBS and re-suspended in appropriate culture medium without antibiotics.

### qPCR

Macrophages were seeded in 6-well plates at a density of 2 × 10^6^ cells per well. RNA was isolated and purified from infected cells using Trizol reagent (Life Technologies) and following the manufacturer protocol. From total RNA 1 μg was used for cDNA synthesis (Superscript^TM^ II reverse transcriptase, Invitrogen), according to the manufacturer protocol. qPCR was performed using SYBR Green PCR Master Mix (Applied Biosystems) and different sets of primers (Table 1) (Eurofins Genomics) at a final concentration of 0.5 μM. The PCR reaction proceeded as follows: 1 cycle of 95 °C for 10 min, followed by 40 cycles of 95 °C for 15 s, 60 °C for 30 sec and, 72 °C for 30 sec. The mRNA expression profiles were normalized with respect to GAPDH (Glyceraldehyde 3-phosphate dehydrogenase). The qPCR was performed using a QuantStudio™ 7 Flex System (ThermoFischer). Relative gene expression was obtained using the instruments software (relative quantity = 2^−∆∆Ct^). The mean of five non-stimulated and uninfected donor samples was taken and used to produce log_2_ ratios with respect to the normalized data from stimulated and/or infected cells. Gene expression heatmaps were generated using TM_4_ MultiExperiment Viewer software and hierarchichal clustering for the gene tree.

### Western Blotting

Macrophages were seeded in 6-well plates at a density of 2 × 10^6^ cells per well. Total proteins were recovered with 200 μl of Laemmli buffer (Sigma-Aldrich). Protein extracts were subjected to electrophoresis in 12% SDS-PAGE gels, transferred to a nitrocellulose membrane and blocked with 0.1% Tween 20, 5% of low fat milk TBS (Tris Buffered Saline). The nitrocellulose membrane was then incubated with primary antibodies specific for human cathepsin S, B and L, and β-tubulin (abcam), overnight at 4 °C. All membranes were washed and incubated with secondary HRP-conjugated antibodies. The bands were visualized with a chemiluminescent HRP-substrate reagent (Merck Millipore) and quantified using ImageJ^59^.

### Enzymatic activity

Macrophages were seeded in 6-well plates at a density of 2 × 10^6^ cells per well, and were recovered with a 5 mM EDTA/PBS solution. Cell lysis and measurement of the enzymatic activity was performed using the Cathepsin Activity Fluorometric Assay kits from Biovision specific for each cathepsin and following the manufacturer’s instructions. Essay specificity was verified by treating the cell lysates with specific inhibitors for each cathepsin, provided in the kit. The fluorescence intensity was measured with a spectrofluorometer, Tecan M200.

### Inhibition of cathepsin activity

Twenty-four hours prior to infection, macrophages cell cultures were treated with 20 μM of inhibitors of cathepsin B, S and L, or with cystatin C (Sigma-Aldrich). E-64d (Sigma-Aldrich), a general irreversible inhibitor of cathepsins, was used at 10 μM after infection due to high cytotoxic effects. Bacteria survival was measured after 24 h of infection.

### RNAi silencing

Silencing of cathepsin B, S and L gene expression was performed according to Troegeler *et al.*^60^ with Biontex K2^®^ Transfection System. Macrophages were first incubated for 2 h with 4 μl/ml of K2 Multiplier reagent in culture medium. Then, they were incubated with the transfection reagent and 100 nM of SMARTpool ON-TARGETplus siRNA (GE Dharmacon) in a ratio of 5 μl_reagent_:1 μg_siRNA_. For 24 h in antibiotic free medium. Transfection medium was then removed and the cells were incubated for 3 days in fresh medium prior to any experiment in order to achieve maximum silencing. The transfection efficiency achieved was approximately 95%, as evaluated by flow cytometry using siGLO Green Transfection Indicator (GE Dharmacon), and the protein and enzymatic activity levels were measured to verify the efficiency of gene silencing.

### Intracellular bacteria survival assay

Macrophages were seeded in 96-well plates at a density of 5 × 10^4^ cells per well. When required, infected cells were lysed in 0.05% IGEPAL CA-630 (Sigma-Aldrich) solution in water, a nonionic, non-denaturing detergent that disrupts eukaryotic cells but does not affects mycobacteria viability, with the goal being to assess the colony-forming microorganisms of viable intracellular bacteria. Serial dilutions of the resulting bacterial suspension were plated in Middlebrook 7H10 agar with 10% OADC (Difco) and incubated for 2–3 weeks at 37 °C before colonies were observable.

### Lentiviral RNAi screen

A subset of the shRNA library from SIGMA was re-arrayed to target cathepsins and cystatins genes. The pLKO.1 shRNA constructs were supplied as glycerol stocks. Minipreps and virus production were performed as described in the TRC online protocols. Briefly, minipreps were performed in 96-well plates (Whatman) and DNAquantified using a Pico green assay (Invitrogen). Lentiviruses were made in 96-well format transfecting 293T cells with 100 ng of the pLKOpuro.1 shRNA plasmid, and the packaging plasmids, CMVdR8.9 and PHCMV- VSV-G, 100 ng and 10 ng respectively. Viruses were collected at 48 h after transfection^61^. THP-1 cells were plated in 96-well round-bottom plates at 5 × 10^4^ cells per well. Ten microlitres of virus plus 8 μg ml^−1^ polybrene was added per well and the plates were spun at 900 × *g*. For 90 min at 37 °C. After spinoculation the supernatants were replaced with fresh media. Forty-eight hours post infection, puromycin was added at a final concentration of 5 μg ml^−1^. After 3 days in puromycin selection, each plate was split into two replica plates. On the sixth day post infection the number of cells per well was enumerated using Guava 5HT flow cytometer and the cells were seeded in order to achieve 5 × 10^4^ cells per well and differentiated into macrophages. Macrophages were infected as previously mentioned and MTB survival was measured after 5 days of infection. A gene was defined as a ‘hit’ when there was an increased or decreased MTB survival >50% and >2 standard deviations from the mean survival of the controls (cells transduced with lentivirus encoding non-silencing scrambled-shRNA were used as controls) in two independent screens. Gene silencing was measured by qPCR.

### Statistical Analysis

Data are presented as mean ± standard deviation. Statistical analysis was made using SigmaPlot 12. Multiple group comparisons were made using ANOVA one parameter tests followed by pairwise comparisons of the groups using Holm-Sidak test. Two group comparisons were made using Student’s t-test. All the prerequisites of the tests were verified. The considered nominal alpha criterion level was 0.05 below which differences between samples were deemed significant.

## Additional Information

**How to cite this article**: Pires, D. *et al.* Role of Cathepsins in *Mycobacterium tuberculosis* Survival in Human Macrophages. *Sci. Rep.* **6**, 32247; doi: 10.1038/srep32247 (2016).

## Supplementary Material

Supplementary Information

## Figures and Tables

**Table 1:** List of qPCR primers.

Target Gene		Target sequence (5′-3′)
Cathepsin B	Forward	CCAGGGAGCAAGACAGAGAC
	Reverse	GAGACTGGCGTTCTCCAAAG
Cathepsin C	Forward	CCCCTACACAGGCACTGATT
	Reverse	CAGCTCAAAGGGGTTGAAAG
Cathepsin D	Forward	GACACAGGCACTTCCCTCAT
	Reverse	CTCTGGGGACAGCTTGTAGC
Cathepsin E	Forward	ACTTGACATCCACCCTCCAG
	Reverse	GAATGCCCCAGCCTAACATA
Cathepsin F	Forward	GCCTGTCCGTCTTTGTCAAT
	Reverse	TGGCTTGCTTCATCTTGTTG
Cathepsin G	Forward	GCCTTTCAGGAAAGATGCAG
	Reverse	CACAAAGTCTTCTCGCACCA
Cathepsin H	Forward	ACGGAGGAGTACCACCACAG
	Reverse	GCAATTCTGAGGCTCTGACC
Cathepsin K	Forward	TTCTGCTGCTACCTGTGGTG
	Reverse	GCCTCAAGGTTATGGATGGA
Cathepsin L	Forward	AGGAGAGCAGTGTGGGAGAA
	Reverse	ATCTGGGGGCCTCATAAAAC
Cathepsin O	Forward	AGTGGGACAAACTCCAGCAC
	Reverse	CCTCTTTGATCCCACCTGAA
Cathepsin S	Forward	TCTCTCAGTGCCCAGAACCT
	Reverse	GCCACAGCTTCTTTCAGGAC
Cathepsin V	Forward	TCCGTGAGCCTCTGTTTCTT
	Reverse	CTAGCCATGAAGCCACCATT
Cathepsin W	Forward	CCACCCCAAGAAGTACCAGA
	Reverse	GTGGCCTTGATCACACCTTT
Cathepsin Z	Forward	GGGAGAAGATGATGGCAGAA
	Reverse	ATAGGTGCTGGTCACGATCC
Cystatin A	Forward	GAGGCTTATCTGAGGCCAAA
	Reverse	TCATTTTGTCCGGGAAGACT
Cystatin B	Forward	TGTCATTCAAGAGCCAGGTG
	Reverse	AGCTCATCATGCTTGGCTTT
Cystatin C	Forward	CCAGCAACGACATGTACCAC
	Reverse	ACAGGTGGATTTCGACAAGG
Cystatin D	Forward	GAAGTTCGGTCGAACCACAT
	Reverse	CCTAGACTTTCCGGCACTTG
Cystatin E/M	Forward	CTACTTCCGAGACACGCACA
	Reverse	GGAACCACAAGGACCTCAAA
Cystatin F	Forward	TGACTTCCAAACCAACCACA
	Reverse	TGCATCCTGGTGTTTGTCAT
Cystatin S	Forward	GCTCCAGCTTTGTGCTCTGCCT
	Reverse	GTCTGCTCCCTGGCTCGCAG
Cystatin SA	Forward	CTGCGGGTGCTACGAGCCAG
	Reverse	GGAGGGAGGGCAGAGTCCCC
Cystatin SN	Forward	TCCCTGCCTCGGGCTCTCAC
	Reverse	ACCCGCAGCGGACGTCTGTA
GAPDH	Forward	AAGGTGAAGGTCGGAGTCAA
	Reverse	AATGAAGGGGTCATTGATGG
