# Supplementary material for: Role of Cathepsins in Mycobacterium tuberculosis Survival in Human Macrophages
Source: Sci Rep. 2016 Aug 30;6:32247. doi: 10.1038/srep32247 (PMC5004184; doi:10.1038/srep32247)
Supplement: Supplementary Information [file srep32247-s1.pdf]

Supplementary Information

Role of Cathepsins in *Mycobacterium tuberculosis* Survival in Human Macrophages

David Pires[1,2], Joana Marques[1], João Palma Pombo[1], Nuno Carmo[1,2], Paulo Bettencourt[1,2#], Olivier Neyrolles[3,4], Geanncarlo Lugo-Villarino[3,4] and Elsa Anes [1,2*]

[1]Research Institute for Medicines, iMed-ULisboa, Faculty of Pharmacy, Universidade de Lisboa, Portugal;

[2]Instituto de Medicina Molecular, Faculdade de Medicina da Universidade de Lisboa, Portugal.

[3]Centre National de la Recherche Scientifique, Institut de Pharmacologie et de Biologie Structurale, Toulouse, France

[4]Institut de Pharmacologie et de Biologie Structurale, Université de Toulouse, Université Paul Sabatier, Toulouse, France

*Corresponding author: Prof. Elsa Anes, eanes@ff.ulisboa.pt.

[#]Present address: Paulo Bettencourt, The Jenner Institute, University of Oxford, Oxford, United Kingdom.

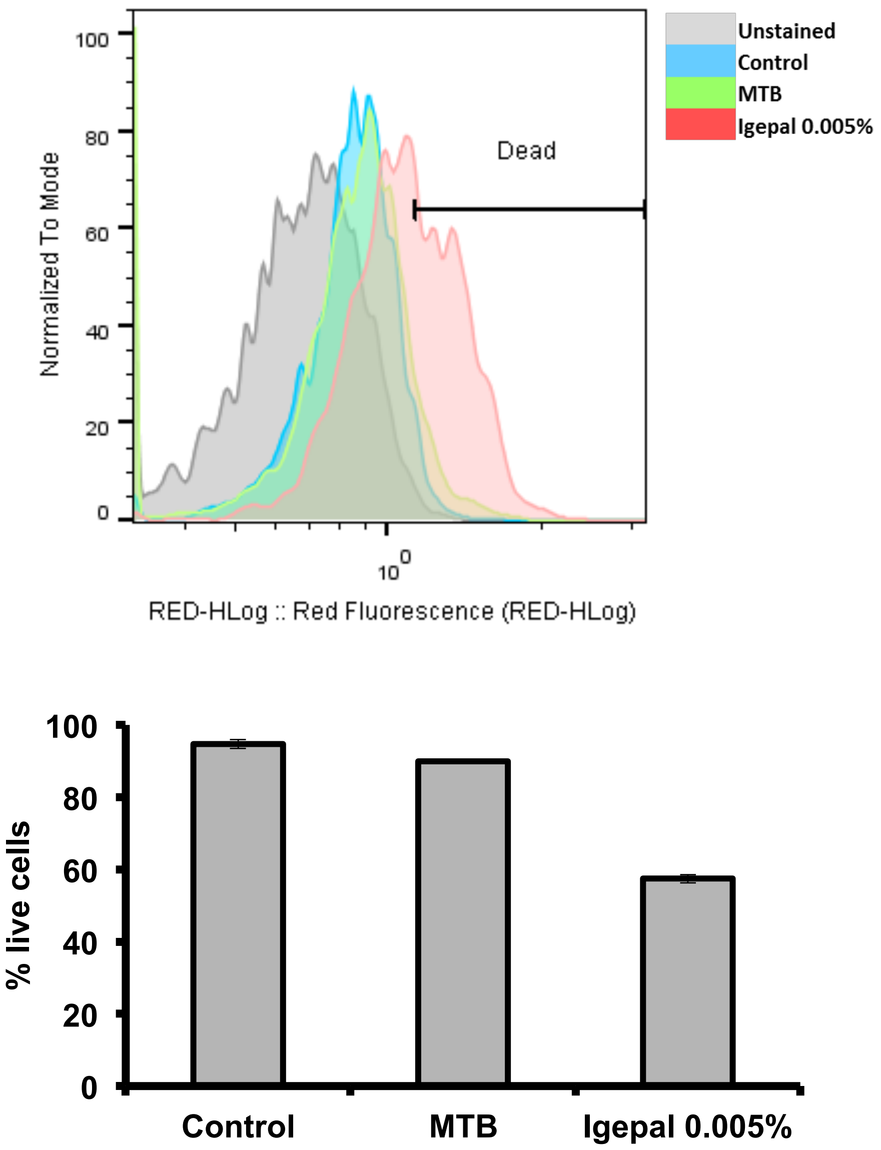

Figure S1. Macrophage viability after 24h of infection with *M. tuberculosis*. Macrophages were stained with propidium iodide and the percentage of stained cells was quantified by flow cytometry. Macrophages treated with Igepal 0.005% for 5 min were used as a positive control for cell death.

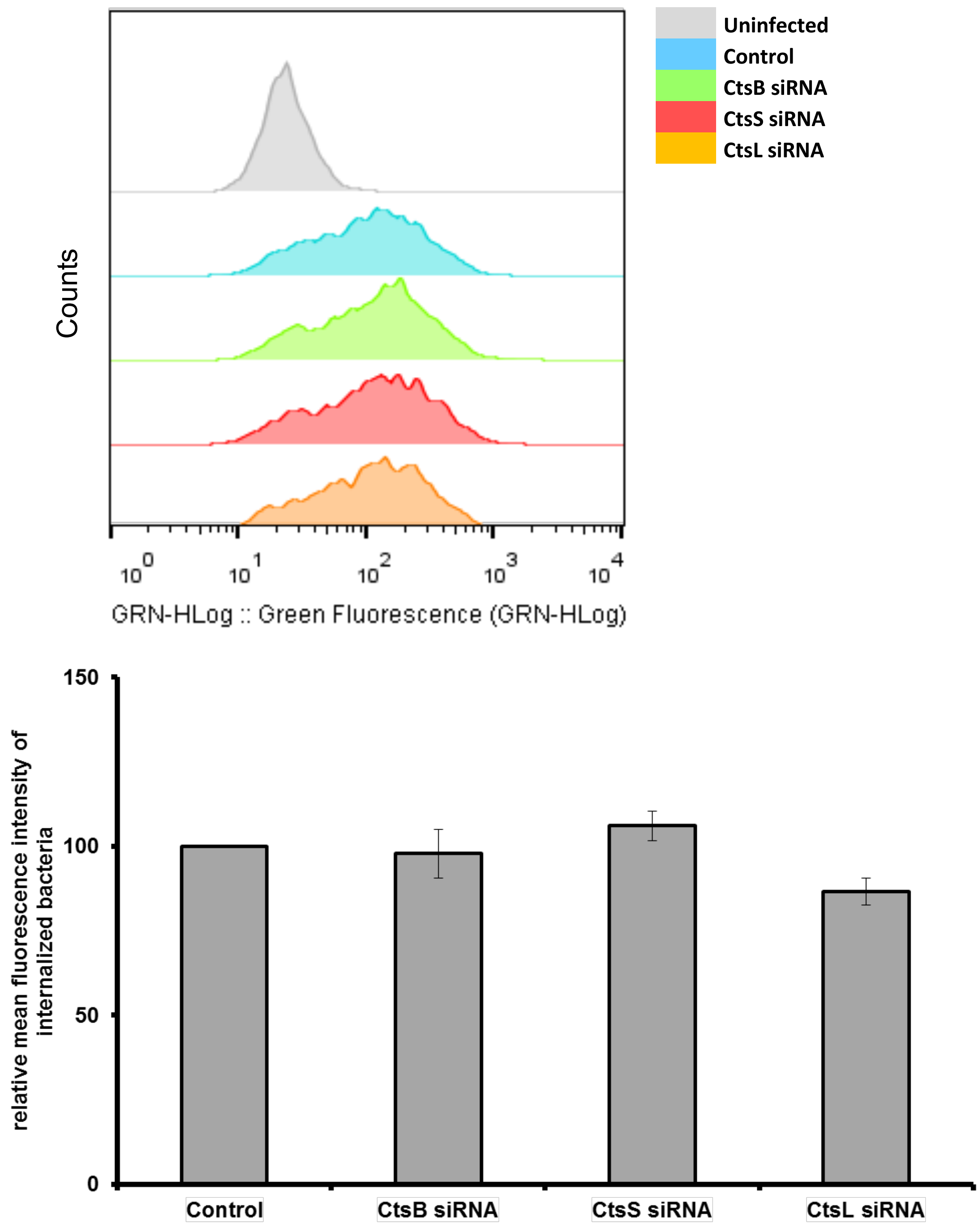

Figure S2. *M. tuberculosis* internalization by primary human macrophages silenced for cathepsins B, S and L. Macrophages transfected with 100nM control, CtsB, CtsS or CtsL siRNA were infected with *M. tuberculosis* expressing GFP and analyzed by flow cytometry after 3h of infection.
